# Identification of DAPK1 as an autophagy-related biomarker for myotonic dystrophy type 1

**DOI:** 10.3389/fgene.2022.1022640

**Published:** 2022-10-20

**Authors:** Min Hu, Meng-Ru Ge, Hong-Xia Li, Bei Zhang, Gang Li

**Affiliations:** Department of Neurology, Shanghai East Hospital, School of Medicine, Tongji University, Shanghai, China

**Keywords:** DAPK1, autophagy-related gene, biomarker, myotonic dystrophy type 1, weighted gene co-expression network analysis

## Abstract

Myotonic dystrophy type I (DM1), a CTG repeat expansion hereditary disorder, is primarily characterized by myotonia. Several studies have reported that abnormal autophagy pathway has a close relationship with DM1. However, the underlying key regulatory molecules dictating autophagy disturbance still remains elusive. Previous studies mainly focused on finding targeted therapies for DM1, but the clinical heterogeneity of the DM1 is rarely addressed. Herein, to identify potential regulator genes related to autophagy and cross-correlation among clinical symptoms, we performed weighted gene co-expression network analysis (WGCNA) to construct the co-expression network and screened out 7 core autophagy-related genes (DAPK1, KLHL4, ERBB3, SESN3, ATF4, MEG3, and COL1A1) by overlapping within differentially expressed genes (DEG), cytoHubba, gene significance (GS) and module membership (MM) score. Meanwhile, we here analyzed autophagy-related molecular subtypes of DM1 in relation to the clinical phenotype. Our results show that three genes (DAPK1, SESN3, and MEG3) contribute to distinguish these two molecular subtypes of DM1. We then develop an analysis of RNA-seq data from six human skin fibroblasts (3 DM1, 3 healthy donors). Intriguingly, of the 7 hallmark genes obtained, DAPK1 is the only confirmed gene, and finally identified *in vitro* by RT-PCR. Furthermore, we assessed the DAPK1 accuracy diagnosis of DM1 by plotting a receiver operating characteristic curve (ROC) (AUC = 0.965). In this study, we first validated autophagy status of DM1 individuals exhibits a clearly heterogeneity. Our study identified and validated DAPK1 serve as a novel autophagy-related biomarker that correlate with the progression of DM1.

## Introduction

As an inherited neuromuscular disease, Myotonic dystrophy type 1 (DM1) is the most prevalent forms of muscular dystrophy in adults (Nguyen and Campbell, 2016). Previous studies in investigation of molecular markers or therapeutic targets in DM1 disease mainly focused on serum protein (Nicoletti et al., 2022), abnormal pre-mRNA splicing (Nakamori et al., 2013) and extracellular RNAs (Koehorst et al., 2020), but not associated with autophagy and molecular subtypes in DM1. In respect of onset, symptoms and severity, DM1 is a highly heterogeneous disorder; also, the progression of disease varies significantly (De Serres-Bérard et al., 2021). The diverse clinical phenotypes of DM1 present a challenge due to the involvement of diversified disease response status. Such heterogeneity in clinical signatures and in assessment of disease severity, make it difficult to develop efficient treatments and, to date, several therapeutics strategies have been applied only to mitigate DM1 progression symptoms. The main obstacle in finding effective therapeutics targets for DM1 might be closely related to clinical heterogeneity. Thus, it is crucial to discover molecular subtypes and signatures in DM1 that correlate to clinical outcomes.

Autophagy is involved in the maintenance of skeletal muscle cellular metabolic balance, thereby playing a critical role for tissue homeostasis (Sciorati et al., 2016). Several studies support a vital role for hyperactivated autophagy response in DM1. The heightened expression levels of autophagy-related genes in skeletal muscle biopsies from DM1 patient and in correlation with DM1 pathogenesis (Fernandez-Costa et al., 2013). Additionally, the reduction of cross-sectional muscle area was observed concomitant with overactivated autophagy in *Drosophila* DM1 model (Bargiela et al., 2015), and the loss of muscle could be rescued by genetic inhibition of the autophagy pathway. Consistent with these observations, cumulative evidence indicates that activation of the autophagic degradation pathway is one of the mechanisms leading to muscle wasting (Fernandez-Costa et al., 2013; Brockhoff et al., 2017; Morriss et al., 2018). A recent study conducted that chloroquine, a chemical inhibitor of autophagy, administration to DM1 models resulted in increased levels of MBNL1 and improved DM1 phenotypes in these models (Bargiela et al., 2019). However, how this autophagy response affects molecular subtypes and signatures of different DM1 patients and how it influences the cross-correlation among clinical phenotype in DM1 is remain unknown. Thus, taken together, further elucidation of the molecular pathways and hub genes involved may provide promising insights for developing DM1 therapies.

In order to interrogate both autophagy status and clinical phenotype correlates of DM1, we performed the weighted gene coexpression network analysis (WGCNA) to identify the key modules highly related to DM1. 7 hyperactivated autophagy hallmark genes of the two key modules were identified from further clinical manifestations analysis. Meanwhile, we first performed molecular subtyping of DM1 patients with different autophagy response status and correlated them with clinical phenotypes. DAPK1 was eventually verified based on a series of bioinformatics analyses and *in vitro*, which expected to provide novel insights into therapeutic targets for the treatment of DM1.

## Materials and methods

### Data collection

RNA-seq data in GSE86356 (Wang et al., 2019) consisted of 55 tibialis muscle samples from 44 Myotonic dystrophy type 1 (DM1) patients and 11 normal individuals were downloaded from Gene Expression Omnibus (GEO) repository. For validation, the dataset GSE47968 (Nakamori et al., 2013) dataset consisting of DM1 (n = 8) patients and normal (n = 8) people’s muscle samples were assessed using GPL5188. Then, a total of 2,209 autophagy-related genes (ATGs) were extracted from NCBI (https://www.ncbi.nlm.nih.gov/), HADb (http://www.autophagy.lu/index.html), AUTOPHAGY DATABASE (http://www.tanpaku.org/autophagy/index.html), GSEA (https://www.gsea-msigdb.org/gsea/index.jsp) and HAMdb (http://hamdb.scbdd.com/) (Supplementary Table S1). The complete workflow is shown in Supplementary Figure S1.

### Cell culture

Human skin fibroblast cell lines from 3 normal individuals and 3 individuals with DM1 were obtained from the biobank of Shanghai East Hospital. Human skin fibroblast cells were grown in DMEM (Life Technologies), NaPyr, GlutaMAX, β-mercaptoethanol and 10% foetal bovine serum (FBS) supplemented medium as previously described (Richner et al., 2015). Cells were collected after 80% confluency with TRIzol reagent and stored at −80°C for RNA-seq and RT-PCR.

### RNA-seq analysis

RNA-seq data (FASTQ files) were mapped against the hg38 genome (GRCh38.p7) reference using Salmon to estimate counts (Patro et al., 2017). Fastp (v0.36) was used to perform quality control and preprocessing (Chen et al., 2018). Salmon estimated counts were converted to the gene level using Tximport (version 3.12) to generate an input file for normalization using DESeq2 (Love et al., 2014), limma (Ritchie et al., 2015) and edgeR (Robinson et al., 2010), respectively. All genes with a cutoff value (FDR < 0.05, |logFC| >1) (Zhang et al., 2021) were considered as differentially expressed genes by DESeq2. Then, we performed similar analyses on our RNA-seq data (human skin fibroblasts). Differential expression analysis in GSE47968 was performed using the limma package (Ritchie et al., 2015) with the same cutoff values as above mentioned.

### Weighted gene coexpression network analysis

Through weighted gene coexpression network analysis (WGCNA), 1888 mapped autophagy-related genes in GSE86356 were subjected to determine coexpressed genes (networkType = ‘signed’) (Langfelder and Horvath, 2008). Pre-processed vst-transformed counts were first checked for missing values and outliers (Supplementary Figure S2A). Meanwhile, the sample dendrogram and trait heatmap are also presented in Supplementary Figure S2B. When the degree of independence was 0.9 (Chai et al., 2021), the lowest power value was selected as the soft threshold power in accordance with a scale-free network. For each module, the module membership (MM) was defined as the correlation of module eigengenes (ME) with gene expression profile, while gene significance (GS) was calculated as the absolute value of the correlation between the ME and DM1.

### Gene ontology enrichment analysis

To decipher the potential biological functional of detected modules in the network, the ClueGO v2.5.8 plugin of Cytoscape v3.9.1 (Bindea et al., 2009) was applied to identify and visualize gene ontology enrichment in key modules, such as biological processes (BP), cellular components (CC) and molecular function (MF). Term enrichment was tested with a Two-sided hypergeometric test, and *p*-values were adjusted by the Bonferroni step-down method. GO terms that were significantly enriched with kappa score (≥0.4).

### Hallmark genes identification

The criteria used for hallmark genes selection were as follows: |GS| > 0.5 & |MM| > 0.8 (Ai et al., 2020), core genes extracted from key modules identified by cytoHubba (Degree method, top 50) and differentially expressed genes in GSE86536. In order to further verify the significance and accuracy of these genes, where the crosstalk genes were identified for further study.

### Identification of autophagy molecular subtypes

Unsupervised hierarchical clustering analysis of the autophagy-related gene expressions in DM1 was performed using the “ConsensusClusterPlus” package (Wilkerson and Hayes, 2010) in R 4.1.

### RNA fluorescent *in situ* hybridization

Human skin fibroblasts were fixed with 4 % PFA (Sigma Aldrich)at RT for 15 min. Slides were prehybridized in 30% volume of formamide and 2× SSC buffer for 10 min at 37°C and hybridized overnight in the solution containing 30% volume of formamide, 2× SSC buffer, 1 mg/ml tRNA, 0.02% BSA, 2 mM EDTA, and Cy3-labeled CAG probe. The incubation was followed by three washes with SSC buffer and DAPI staining for 15 min. After DAPI staining, the fluorescent signals were analyzed using the Laser Scanning Confocal Microscopy under the same brightness and exposure time.

### RT-PCR

Relative expression of DAPK1 was examined under semiquantitative conditions described previously by using SYBR Green (Dastidar et al., 2018). The sequences of the primers are as follows. For hDAPK1, the forward primer is 5′- ACG​TGG​ATG​ATT​ACT​ACG​ACA​CC-3′ and the reverse primer is 5′- TGC​TTT​TCT​CAC​GGC​ATT​TCT-3′, hGAPDH housekeeping gene (5′- ATG​ACA​TCA​AGA​AGG​TGG​TG-3′ and 5′- CAT​ACC​AGG​AAA​TGA​GCT​TG-3′) as control for normalization. The experiments were repeated 3 times for each analyzed gene, and the average values were presented.

### Gene set enrichment analysis

GSEA is primarily applied for interpreting significant cumulative changes of gene expression in RNA-seq and microarray data based on related biological information. The ATGs was used for GSEA to assess group that were significantly with autophagy. Briefly, gene set satisfying normalized *p*-value <0.05, |NES| ≥ 1.0 and false discovery rate (FDR) < 0.25 (Oshi et al., 2021) were sets as cut-off criteria in GSEA v4.2.2 software.

### Statistics analysis

The R software environment (v4.1.0) was used to perform statistical analyses. Mann–Whitney test was used to assess the differences between the two groups. *p*-values < 0.05 was granted statistically significant. ‘pROC’ package was performed to analyse the data and draw the receiver operating characteristic curve (ROC).

## Result

### Weight gene Co-expression network construction and key module identification

**FIGURE 1:**
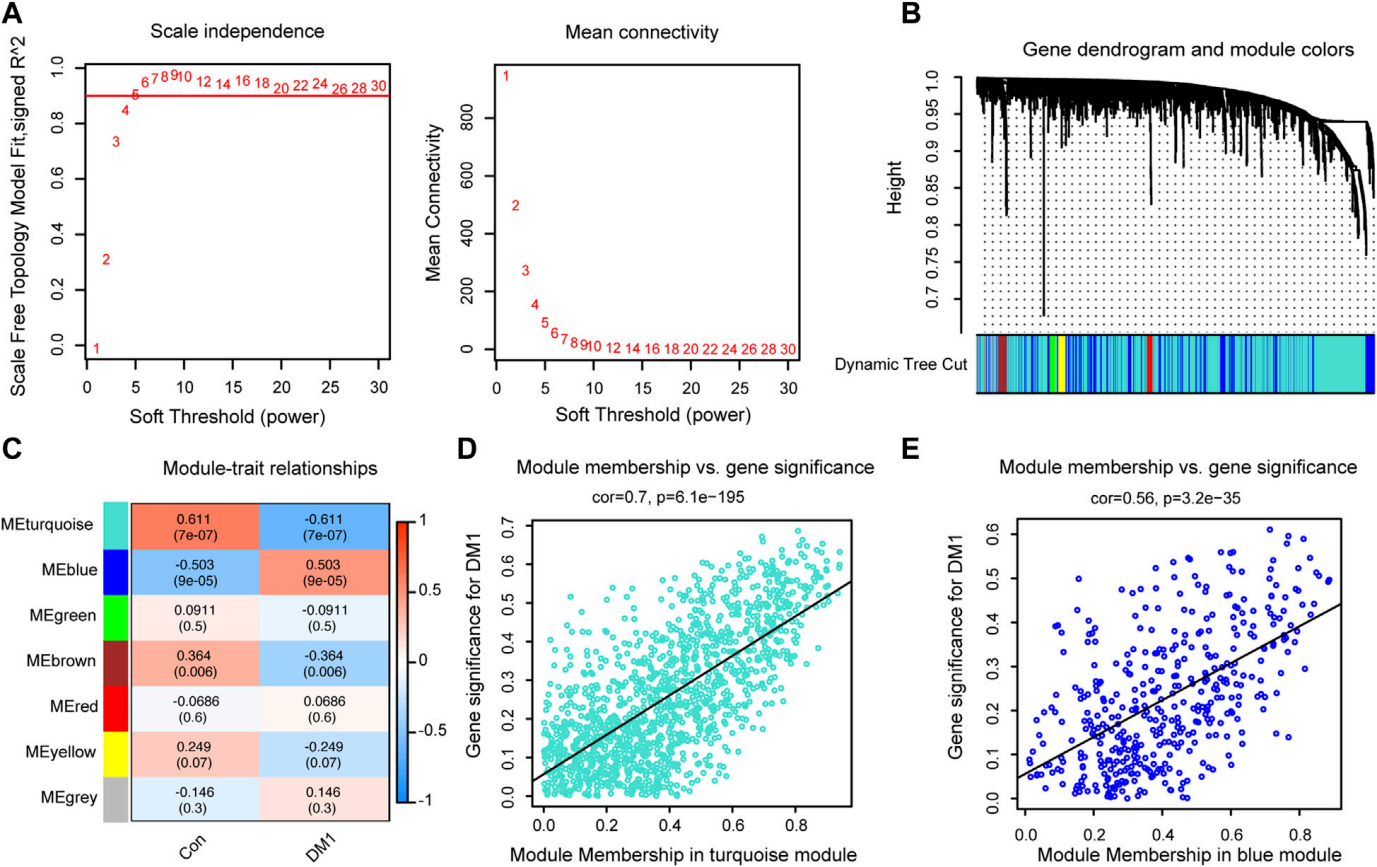
Identification autophagy-related key modules via Weighted gene co-expression network analysis (WGCNA). **(A)** Diagrams showing analysis of network topology based on various soft-thresholding powers. **(B)** Gene clustering dendrogram and module assignment by WGCNA. **(C)** Module-trait relationships between seven key modules and clinical traits. Scatterplot of module membership (MM) versus gene significance (GS) for DM1 in the network of turquoise **(D)** and blue modules **(E)**. Con, control; DM1, myotonic dystrophy type 1.

To further clarify autophagy-related signature genes that have similar expression profiles in response to myotonic dystrophy type 1 (DM1), we screened out 1888 autophagy-related genes from control (n = 11) and (n = 44) samples in GSE86356 to undergo weighted gene coexpression network analysis (WGCNA). A scale-free network (scale-free R2 = 0.93, slope = −1.7, Supplementary Figure S3A) was constructed with a soft thresholding at 5, and a correlation coefficient threshold set at 0.9 (Figure 1A). This consensus WGCNA analysis a total of 7 different modules, painted with different colors, where each module is a cluster of co-expressed genes (Figure 1B). By relating the module eigengene to DM1 genotype, the blue module showed a high positive correlation with DM1 phenotype and turquoise module was significantly negatively correlated with DM1 disease (Figure 1C, Supplementary Figure S3B). The turquoise (cor = 0.7, *p* = 6.1e−195, Figure 1D) and blue (cor = 0.56, *p* = 3.2e−35, Figure 1E) modules composed of mRNAs showed the highest gene significance with DM1 cluster and, hence, were selected for further analysis. The relationship between module membership (MM) and gene significance (GS) in other modules were also shown in Supplementary Figures S3C–G.

### Functional enrichment analysis of genes in the key modules

**FIGURE 2:**
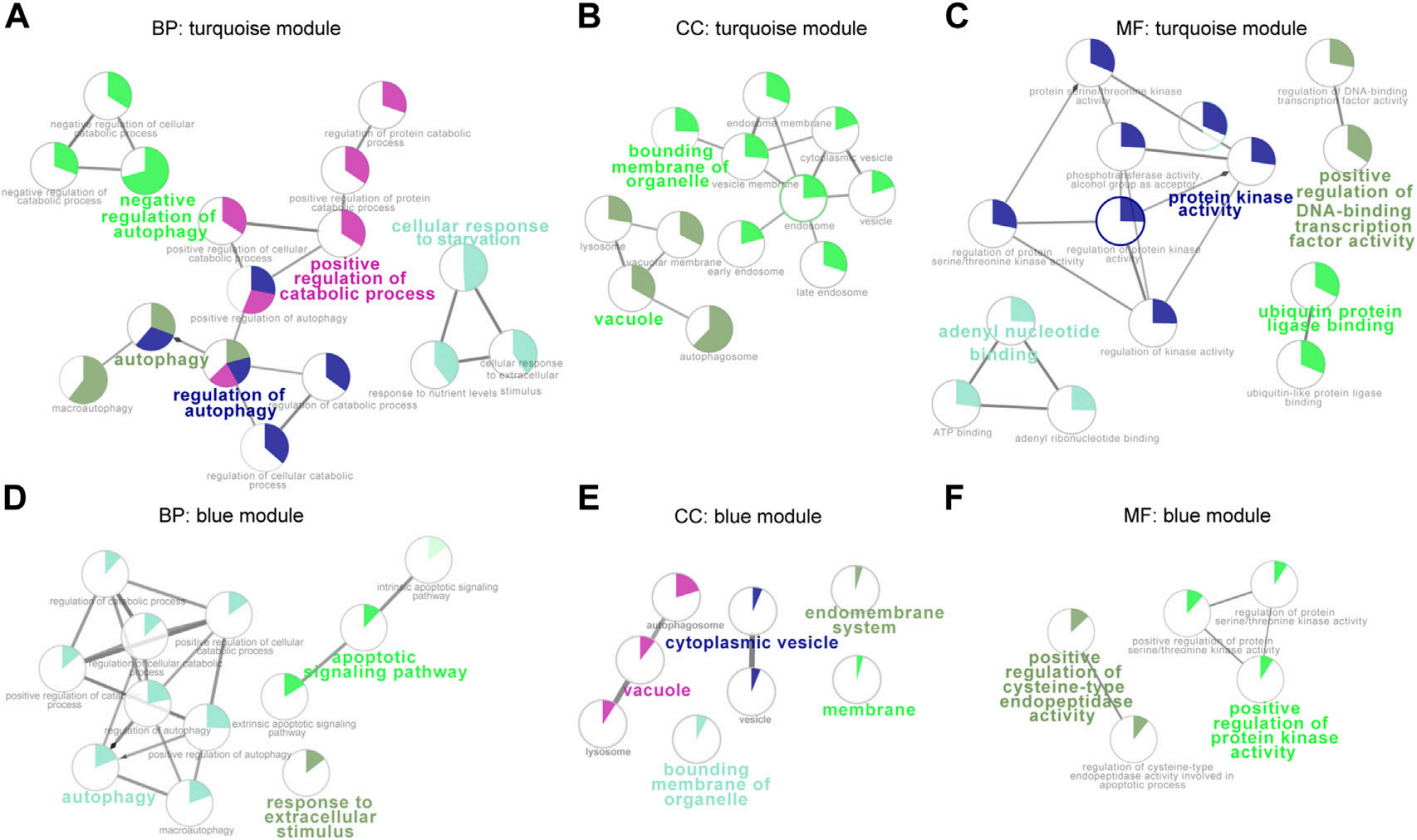
Functional enrichment analysis of the blue and turquoise modules. Over-represented GO/pathway terms were evaluated based on kappa value. The sector size of each category within a pie chart indicates the number of enriched genes. GO/pathway terms are represented as nodes, and the node size highlight the term enrichment significance. Genes in turquoise and blue modules were annotated by biological processes **(A**,**D)**, cellular components **(B**,**E)**, and molecular functions **(C**,**F)**. BP, biological processes; CC, cellular components; MM, molecular functions.

To better understand gene functions of the key modules, we performed Gene Ontology (GO) analysis using clueGO, which mainly includes biological processes (BP), molecular functions (MF), and cellular components (CC). Enrichment of Gene Ontology (GO) BP terms for the turquoise module were mainly focused on autophagy, regulation of autophagy, positive regulation of catabolic process, and cellular response to starvation (Figure 2A), whereas the blue module genes were generally enriched in autophagy, response to extracellular stimulus and apoptotic signaling pathway (Figure 2D). Cellular components GO terms of turquoise module genes showed that most of these mRNAs belong to vacuole and bounding membrane of organelle (Figure 2B), whereas the blue module genes were enriched in membrane proteins such as membrane, the bounding membrane of organelle, vacuole, cytoplasmic vesicle, and endomembrane system (Figure 2E). In terms of molecular functions enrichment, turquoise module genes were mainly focused on protein kinase activity, adenyl nucleotide binding, ubiquitin protein ligase binding and positive regulation of DNA-binding transcription factor activity (Figure 2C), blue module genes were mainly involved in positive regulation of cysteine-type endopeptidase activity and positive regulation of protein kinase activity (Figure 2F).

### Identification of hallmark genes in external datasets

**FIGURE 3:**
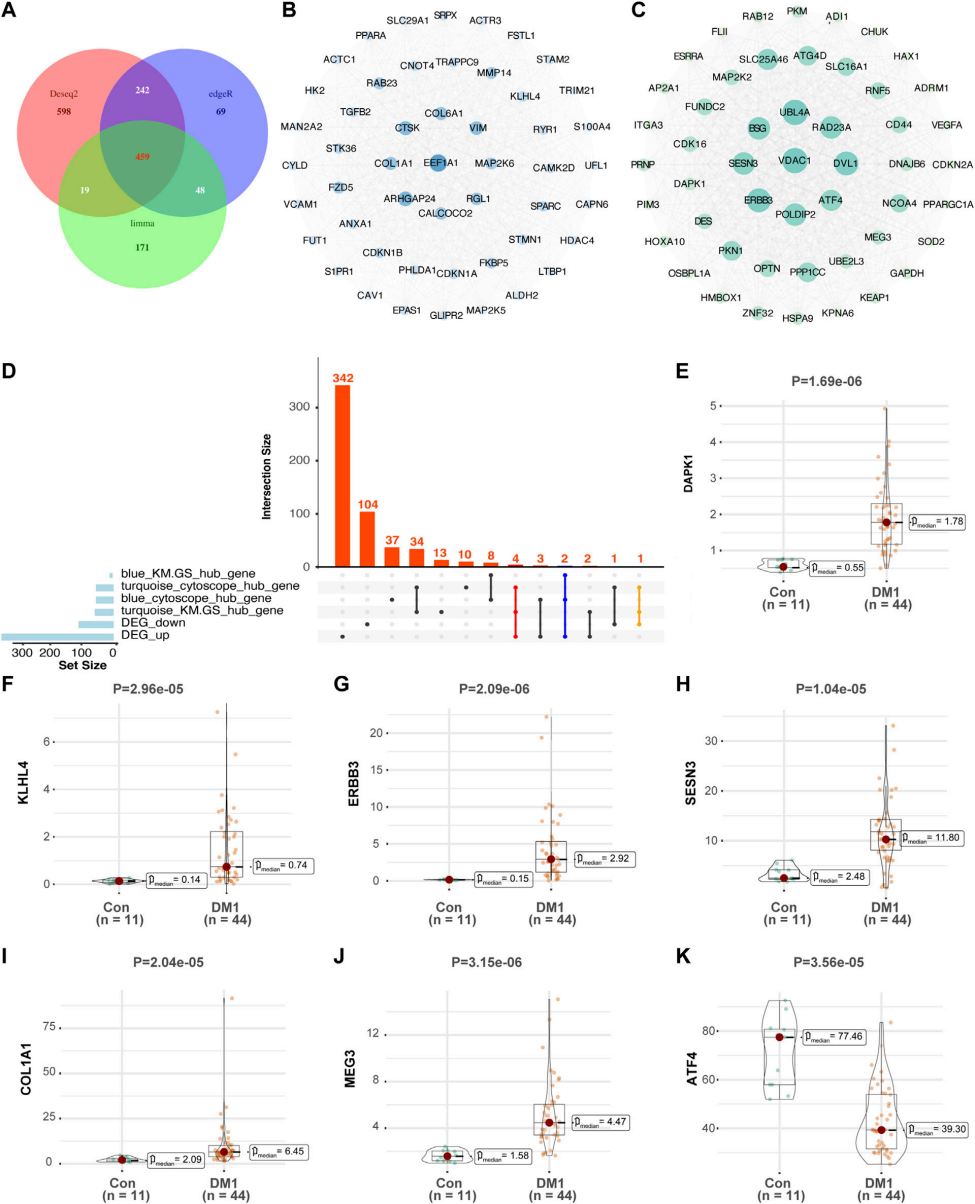
Expression of autophagy-associated hallmark genes in GSE86356 dataset. **(A)** Intersection of three parametric ways (DESeq2, edgeR and limma-voom) of differential expression genes by Venn diagram. According to the degree method, top 50 hub genes were selected by CytoHubba between blue **(B)** and turquoise modules **(C)**, Gene terms are represented as nodes, and the node size indicates weighted degree score. **(D)** The upset plot summarizes overlap of genes between each gene set. **(E**–**K)** Expression levels of DAPK1, KLHL4, ERBB3, SESN3, COL1A1, MEG3, and ATF4 were significantly increased in DM1 patients, whereas the expression of ATF4 was significantly decreased in DM1 patients. Con, control; DM1, myotonic dystrophy type 1.

In differential expression analysis (44 DM1 vs. 11 normal samples), a total of 459 differentially expressed genes were obtained was assessed using three parametric methods including DESeq2, edgeR and limma-voom (Figure 3A), of which 353 genes were upregulated and 106 genes were downregulated in the DM1 samples compared with normal samples in GSE86356 (Supplementary Table S2). To better understand the influence of autophagy-related genes in DM1, we screened the top 50 core genes from cytoHubba (Figures 3B,C), then intersected with DEG and |MM|>0.8 & |GS|>0.5 genes respectively, retained co-expressed genes were considered as hallmark genes. As a result, 7 genes were identified as hallmark genes (DAPK1, KLHL4, ERBB3, SESN3, ATF4, MEG3, and COL1A1), and the upset plot is shown in Figure 3D. Notably, ATF4 was significantly decreased in DM1, and the expression level of other hallmark genes was higher in the DM1 group than the control group (Figures 3E–K). In a confirmatory analysis, four hallmark genes were detected in the GSE47968 microarrays matrix, and the expression trend of DAPK1, ERBB3 and SESN3 were consistent with GSE86356, but not COL1A1 (Supplementary Figures S4A–D).

### New molecular subgroups in DM1

**FIGURE 4:**
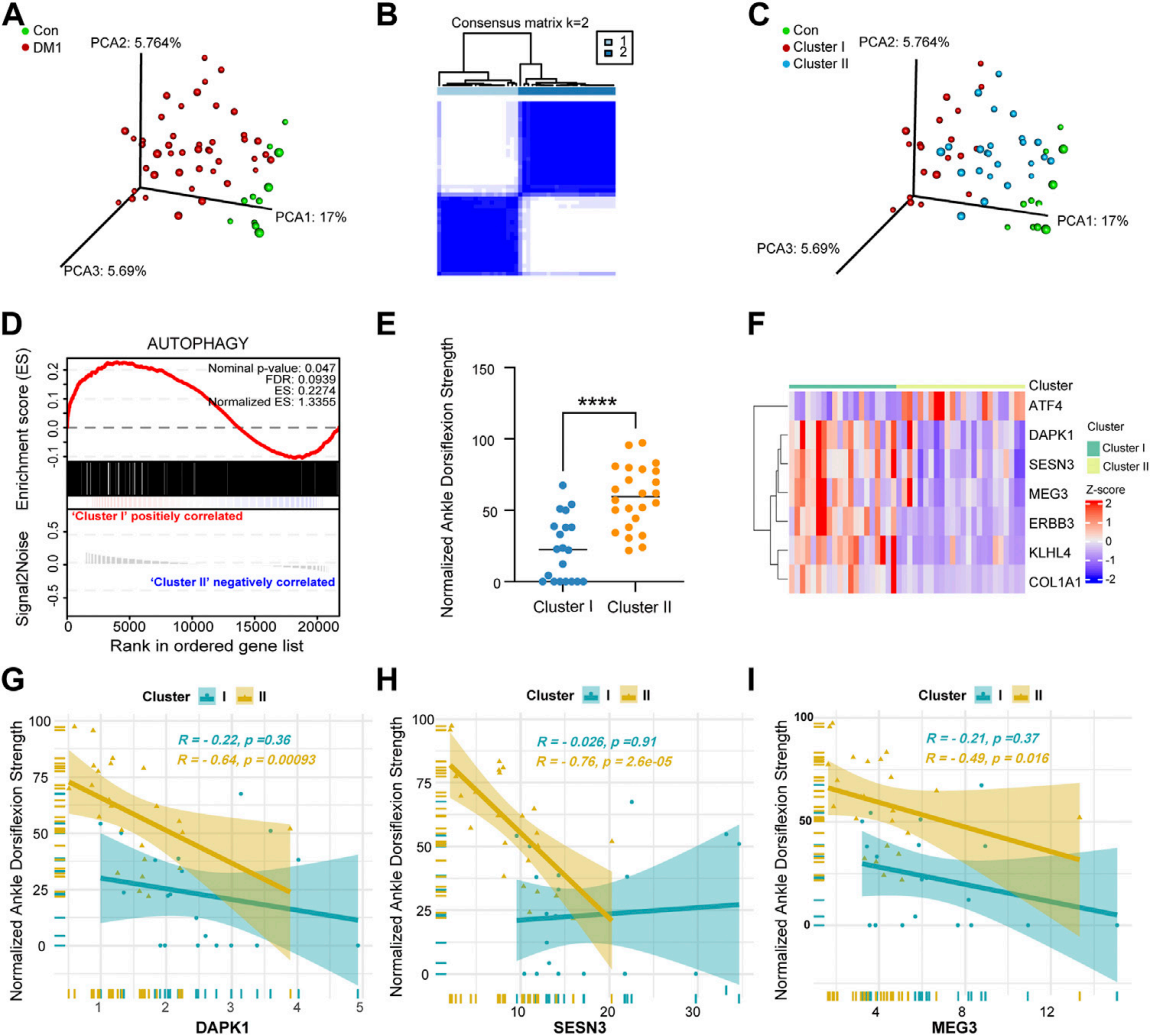
Identification of molecular subgroups in DM1 based on autophagy-related gene sets. **(A)** Principal component analysis (PCA) based on 1,888 ATGs showing a higher heterogeneity between DM1 and control group in GSE86356. **(B)** The two consensus molecular subgroups were identified by consensus clustering analysis in DM1. **(C)** PCA plots of different group based on 1,888 ATGs colored by group types. **(D)** The GSEA plot shows significant enrichment (NES = 1.3355, FDR = 0.0939) of ATGs in DM1 vs. Con. **(E)** The scatter plots showing the differential clinical signatures of genes between the two molecular subgroups. **(F)** Heatmap of 7 hallmark genes between cluster I and cluster II. **(G**–**I)** The line chart of correlation coefficients performed that the gene expression of DAPK1, SESN3 and MEG3 relative to normalized ankle dorsiflexion strength between the two subgroups of DM1. Con, control; DM1, myotonic dystrophy type 1. ^****^ *p* < 0.0001.

DM1 is known for its clinical heterogeneity, we first investigated the autophagy gene set of patients in our cohort for distinct molecular signatures. According to the expression of mapped 1,888 ATG in GSE86356, the principal component plot analysis (PCA) of these genes highlights the heterogeneity within DM1 patients, with one group showing clear differences compared to controls and the other showing no such separation (Figure 4A). Subsequently, the autophagy molecular subtypes were performed by the “ConsensusClusterPlus” package. As is shown in Figure 4B, K = 2 could make the subtypes independent of each other which was confirmed by PCA (Figure 4C). Additionally, autophagy-related genes were used as gene signatures for Gene Set Enrichment Analysis (GSEA) (Subramanian et al., 2005) and showed significant enrichment in cluster I compared to cluster II (Figure 4D), of which cluster I represented a more severe clinical phenotype than cluster II (Figure 4E). Then, the expression levels of 7 hallmark genes in two clusters were visualized by heatmap (Figure 4F) and boxplot charts (Supplementary Figures S5E–K). We found that the expression of three autophagy-related genes (DAPK1, SESN3, and MEG3, Figures 4G–I) negatively correlated with normalized ankle dorsiflexion strength in cluster II, but not KLHL4, ATF4 and COL1A1 (Supplementary Figures S5B–D). Meanwhile, the normalized ankle dorsiflexion strength showed a strong negative correlation with ERBB3 gene (Supplementary Figure S5A) expression in both two molecular clusters. These results support the concepts that autophagy response status from different DM1 subjects exhibits a clearly heterogeneity. Furthermore, the receiver operating characteristic curve (ROC) analysis illustrated that the identified the three hallmark genes potentially serve as good biomarkers to discriminate the two subgroups (Supplementary Figure S5L).

### Identification of autophagy-related hub gene in DM1

**FIGURE 5:**
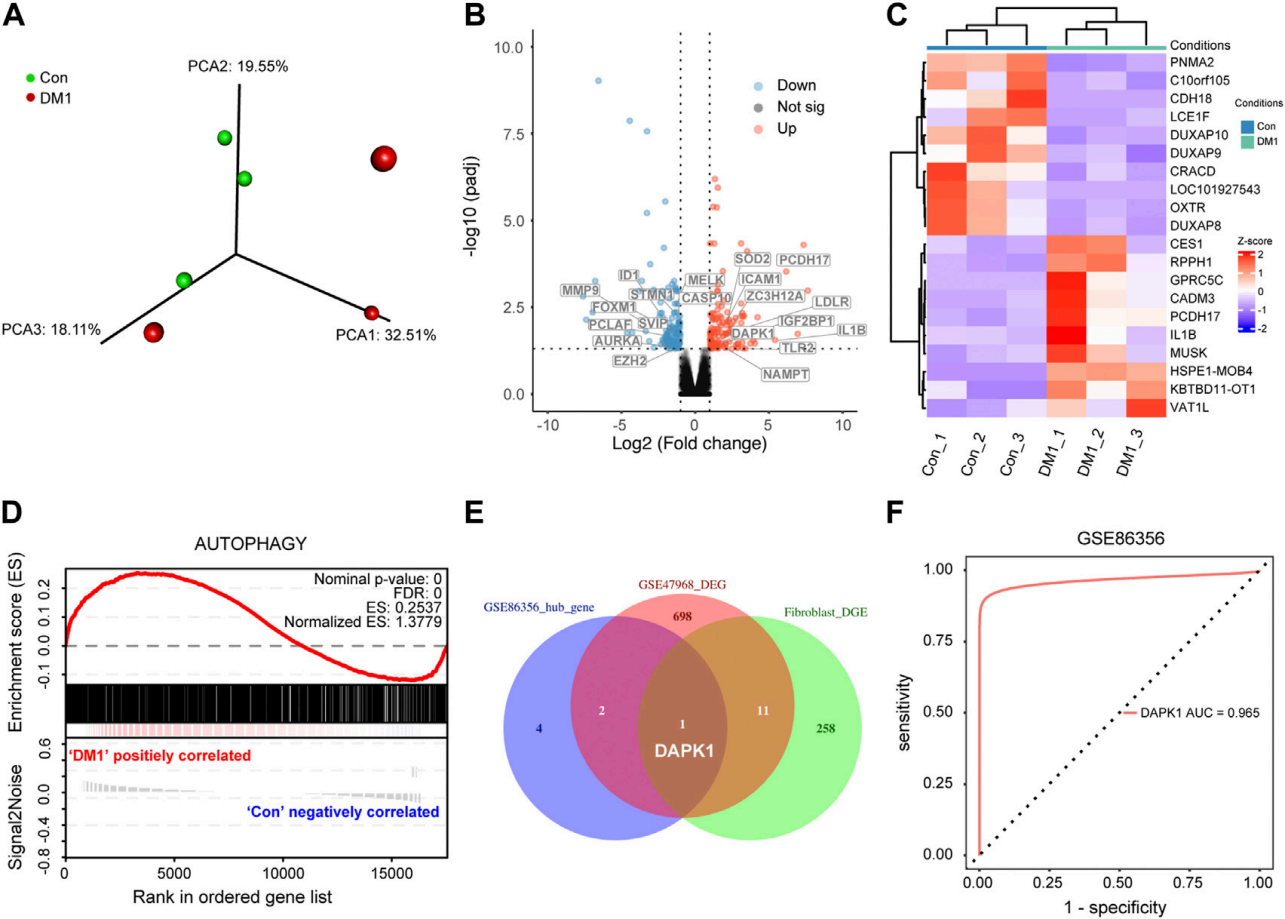
Screening of Autophagy-related hub gene analysis and visualization. **(A)** Principal component plot analysis (PCA) of the human skin fibroblasts profiles obtained from the healthy subjects and DM1 patients. Red and green colors represent DM1 and control group, respectively. **(B)** The top 20 differentially ATG were labeled in the heatmap, with red dots representing significantly up-regulated genes, blue dots representing significantly down-regulated genes. **(C)** Transcripts differentially regulated between DM1 and control human skin fibroblasts. **(D)** Gene set enrichment analysis (GSEA) results show significant enrichment of autophagy in DM1 based on the autophagy-related genes signature. **(E)** Venn diagram which identified the intersection expressed genes among three gene sets. **(F)** Receiver operating characteristic (ROC) analysis was used to evaluate the diagnostic performance of DAPK1 for DM1 in GSE86356. Con, control; DM1, myotonic dystrophy type 1; DEG, differential expression genes.

To further explore the role of autophagy in myotonic dystrophy type 1 (DM1), we sequenced and analyzed normal and DM1 human skin fibroblasts. Gene differential expression analysis between DM1 and normal controls identified a total of 270 genes that were differentially expressed, of which 142 genes were down-regulated and 128 genes were up-regulated (FDR < 0.05, |logFC| >1, Supplementary Table S3). The PCA plot of these expressed genes was performed to show clustering of DM1 and control (Figure 5A) (Panwar et al., 2021). Meanwhile, we included the significantly up-regulated and down-regulated genes to generate a volcanic map to demonstrate their relative expression levels (Figure 5B). Moreover, the top 20 DEG are shown in the heatmap (Figure 5C). Additionally, the gene set enrichment analysis (GSEA) (Mootha et al., 2003; Subramanian et al., 2005) based on autophagy-related signature genes demonstrated that the autophagy pathway was obviously enriched in DM1 group (Figure 5D), which were further verified in GSE47968 database (Supplementary Figure S4F). GSE47968 differential expression genes was provided in Supplementary Table S4. Subsequently, the intersection gene, DAPK1, between DEG (GSE47968 and human skin fibroblasts) and hallmark genes were extracted as a hub gene and used to identify potential prognostic genes for DM1 (Figure 5F), which were visualized as a Venn diagram (Figure 5E). Overall, these results reveals that DAPK1 play a crucial role in DM1 patients with heterogeneous autophagy status and can act as a biomarker for DM1.

### Validation the hub gene *in vitro*

**FIGURE 6:**
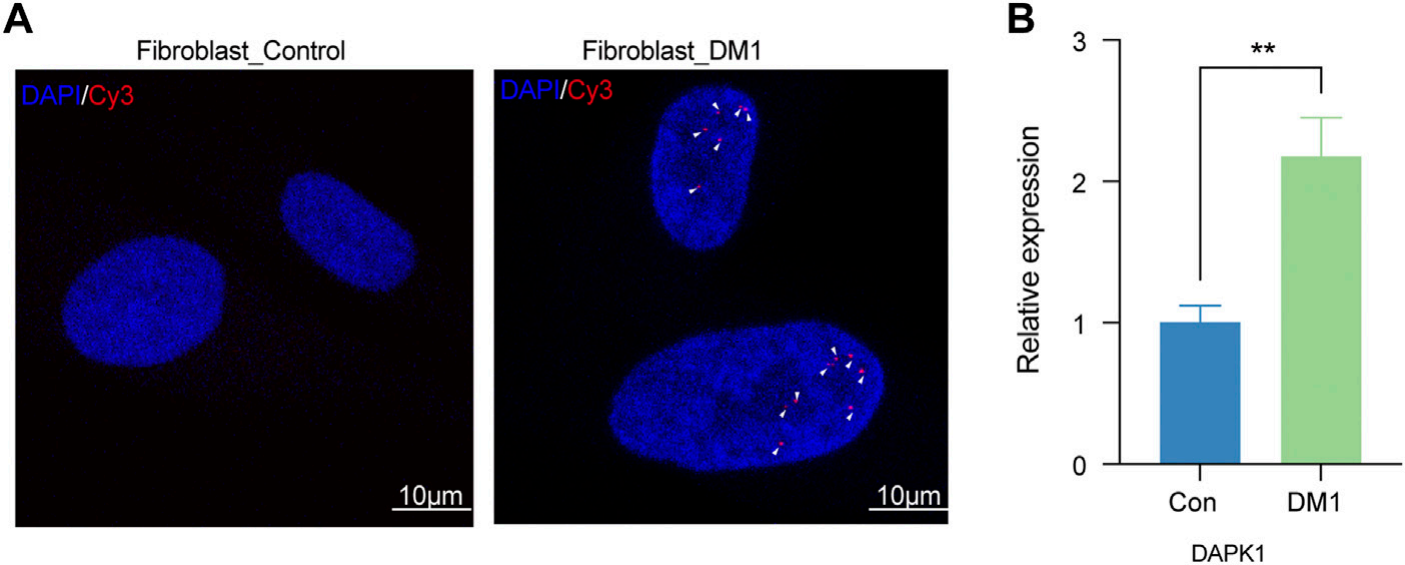
Validation of DAPK1 *in vitro*. **(A)** Representative image of Myotonic dystrophy type 1 (DM1) and healthy control fibroblast stained for RNA foci by fluorescent *in situ* hybridization (FISH). Arrowheads indicate multiple RNA foci (red color) in nuclei of DM1 fibroblast (scale bar = 10 μm). Nuclei were counter-stained with DAPI (blue color). **(B)** The difference expression levels of DAPK1 by RT-PCR between DM1 (*n* = 3) and control fibroblasts (*n* = 3), ^**^ *p* < 0.01.

To further verify the expression of DAPK1 gene in DM1, we performed relevant *in vitro* experiments. The characteristic ribonuclear foci (Batra et al., 2021), a pathology mark of Myotonic dystrophy type 1 (DM1), were also detected in the human skin fibroblasts. As expected, no ribonuclear foci were detected in the skin fibroblast derived from a healthy donor (Figure 6A). RT-PCR results showed that the level of DAPK1 in DM1 skin fibroblast was significantly higher than normal skin fibroblast (Figure 6B). In view of above results, DAPK1 may be a potential critical regulator of autophagy processing in DM1.

## Discussion

Studies have long clearly suggested that imbalances in autophagy changes are the underlying progression mechanisms of the Myotonic dystrophy type 1 (DM1) (Sciorati et al., 2016). However, there is a lack of highly specific diagnostic and therapeutic biomarkers for different molecular subgroups in DM1. With this in mind, we performed weighted gene coexpression network analysis (WGCNA). To systematically evaluate the autophagy-related gene co-expression patterns in DM1. We discovered 7 modules, among which the turquoise and blue modules were markedly aligned with DM1. Seven hallmark genes (DAPK1, KLHL4, ERBB3, SESN3, ATF4, MEG3, and COL1A1) were selected from the two key modules by multiple bioinformatics analyses from GSE86356 dataset. In addition, we further investigated the molecular subgroups of DM1 based mainly on different autophagy status and associated with clinical phenotypes. A major contribution of our study is that we first identified critical gene, DAPK1, which correlate strongly with different autophagy response in DM1 molecular subgroups and evaluated the usefulness for the diagnosis of the DM1.

DM1 is heterogeneous in nature involving interpatient variability in disease progression, autonomic nervous system symptoms, endocrine, and cardiac involvement (Day and Ranum, 2005). Therefore, determining the molecular subtypes of DM1 may lead to novel personalized treatment options. With this in mind, we performed a clustering analysis of the samples in GSE86356 by ‘ConsensusClusterPlus’ R package based on the autophagy-related genes and further clustered the DM1 patients into two distinct groups, throughout this work. Meanwhile, we found that autophagy remains pathologically overactivated in cluster I compare to the cluster II subgroup, and the heightened autophagy status in DM1 shown a more serious clinical phenotype. In general, expression levels of the 7 hallmark genes (DAPK1, KLHL4, ERBB3, SESN3, ATF4, MEG3, and COL1A1) are significantly differentially expressed between cluster I and cluster II within DM1 patients. Three (DAPK1, SESN3, and MEG3) of these 7 genes were negatively correlated with clinical phenotypes in cluster I group, but not in the cluster II group. The area under the curve (AUC) of four hallmark genes exceeded 0.77 by the receiver operating characteristic curve (ROC) analysis. Combined with the above analysis, different subjects exhibit a clearly heterogeneous autophagy response status and we speculated that three hallmark genes could be utilized to distinguish two new molecular subgroups.

Additionally, GSEA analysis showed that autophagy pathway was also enriched in DM1 within the human skin fibroblasts and GSE47968 database, in accordance with the previous studies (Bargiela et al., 2015). In our study, the cored autophagy-related biomarker, DPAK1, was extracted from differential analysis in skin fibroblasts, along with overlapping the differential expression genes in GSE47968 and 7 hub gene in GSE86356. DAPK1, a calcium/calmodulin-dependent serine/threonine kinase, is an important regulator of cell death and autophagy (Maiuri et al., 2009; Oikonomou et al., 2016; Singh et al., 2016; Farag and Roh, 2019). Current research on DAPK1 mainly focuses on cancers (Movahhed et al., 2022), Alzheimer’s disease (Chen et al., 2020; Qi et al., 2020; Wang et al., 2021), stroke (Wang et al., 2017), Parkinson’s disease (Dachsel et al., 2011) and epilepsy (Gan et al., 2021). However, the knowledge regarding the role of DAPK1 in DM1 disease remains unknown. Previous studies have suggested calcium homeostasis is altered in DM1 muscle, and influences disease mechanisms and progression (Kimura et al., 2005; Vihola et al., 2013; Vallejo-Illarramendi et al., 2014; Crawford Parks et al., 2017). Furthermore, disrupted calcium homeostasis has been recently reported in DM1 mouse models. It was shown that hampered calcium uptake by the sarcoplasmic reticulum in DM1 skeletal muscle leads to a highly increased level of cytoplasmic calcium concentration. In turn, elevated calcium levels increase binding with calmodulin (CaM) and trigger multiple calcium-dependent signalling pathways (Crawford Parks et al., 2017). Noteworthily, DAPK1 can be activated by the overloading calcium influx, and its activity can be regulated by cellular Ca^2+^ levels (Tang et al., 2018). Here, we systematically performed bioinformatic analysis to identify DAPK1 that is involved in the autophagy-related pathogenesis of DM1. The ROC curve showed that DAPK1 was of high diagnostic value for DM1 and the RT-PCR analysis further validated that DAPK1 expression was significantly increased in DM1. Thus, we speculated that the increased cellular calcium levels might trigger DAPK1-dependent autophagy signalling pathways in DM1. Collectively, it would be important to verify the biological roles of DAPK1 in the progression of DM1.

Myotonic dystrophy type 1 is a multisystemic disorder with variable organ clinical features (Johnson et al., 2021). In the current study, due to the limited clinical data, there is still a need to confirm the cross-correlation between DAPK1 and other clinical manifestations in future DM1 clinic studies. Meanwhile, we plan to focus on exploring the further mechanism of DAPK1 *in vivo* studies for DM1.

## Conclusion

In conclusion, we found that an autophagy-based molecular heterogeneity in transcriptome profiles of DM1 patients that correlated with severity of clinical manifestations. DAPK1, was screened out with the values of an autophagy-related biomarker for DM1, and further validated *in vitro*. Our findings contribute to a comprehensive understanding of DM1 clinical heterogeneity from the perspective of autophagy response status. Treatment targeting DAPK1 and its downstream signaling pathways may be a novel therapeutic target for DM1.

## Data Availability

The datasets presented in this study can be found in online repositories. The names of the repository/repositories and accession number(s) can be found below: https://www.ncbi.nlm.nih.gov/geo/, GSE211497.
