# Supplementary material for: Identification of DAPK1 as an autophagy-related biomarker for myotonic dystrophy type 1
Source: Front Genet. 2022 Oct 20;13:1022640. doi: 10.3389/fgene.2022.1022640 (PMC9634726; doi:10.3389/fgene.2022.1022640)
Supplement: Supplementary file 5 [file DataSheet1.PDF]

Supplementary Figure

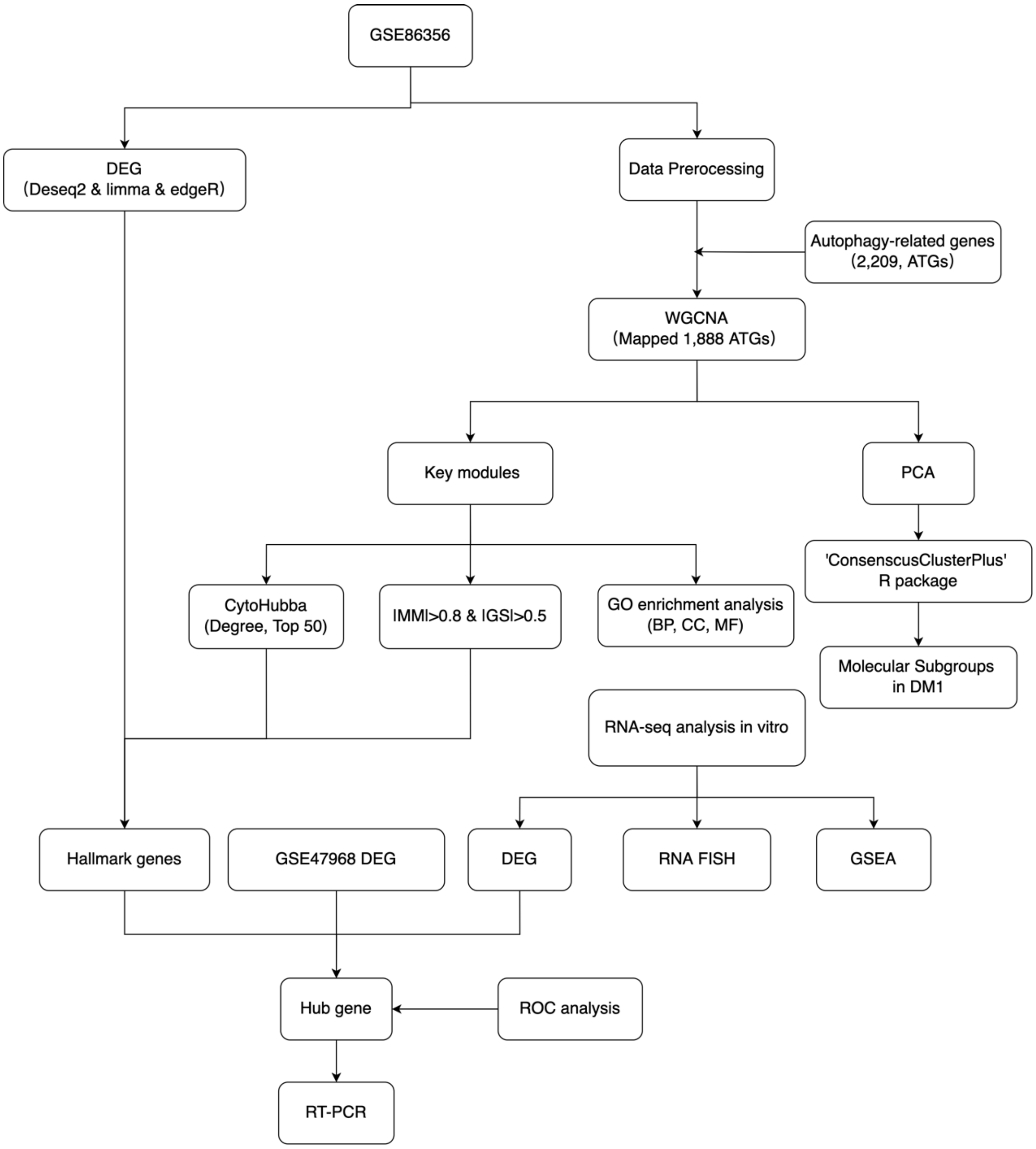

Supplementary Figure 1. Summary of the workflow for the study.

Supplementary Figure 2

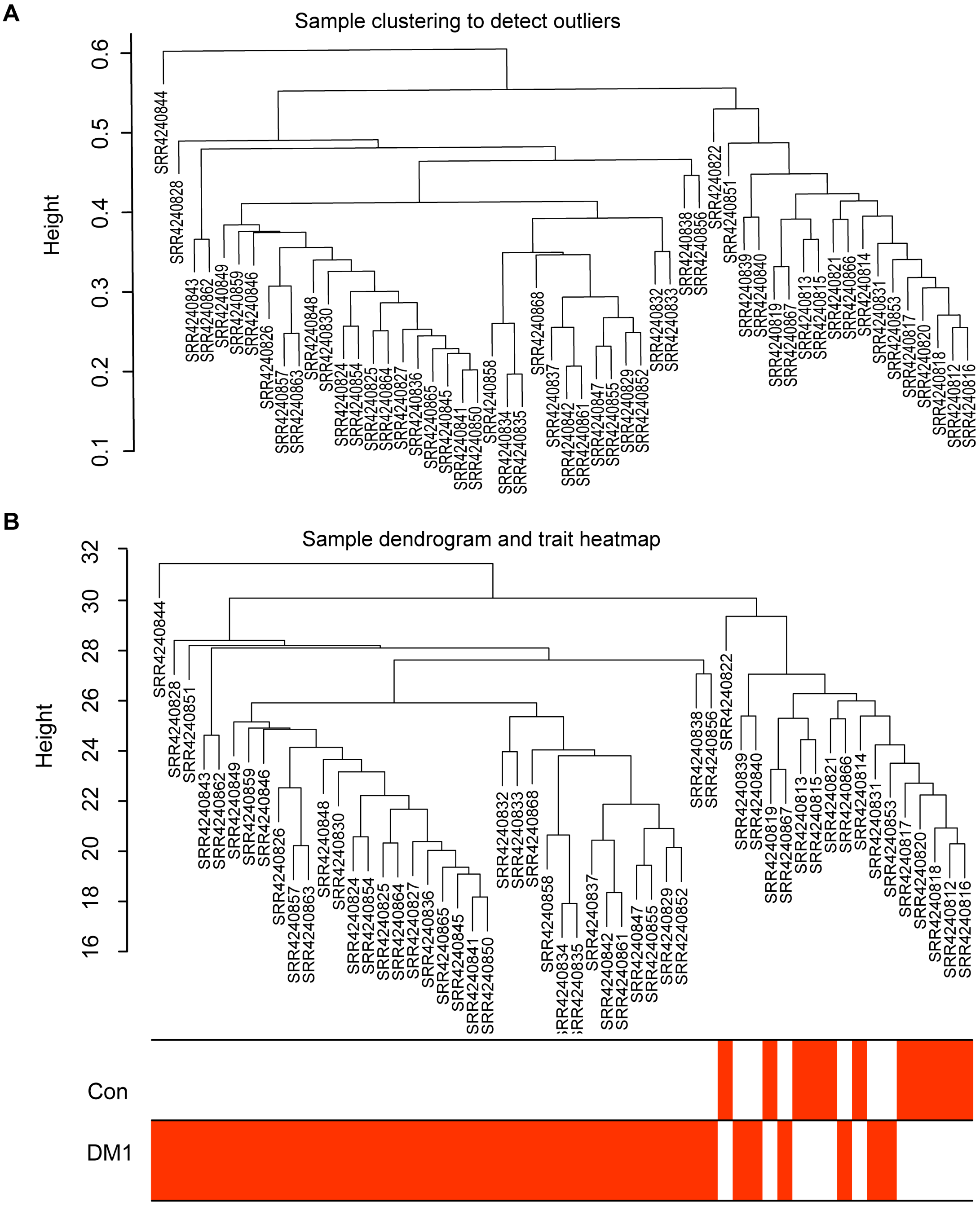

Supplementary Figure 2. Sample clustering. (A) Sample clustering analysis was performed to detect outliers in GSE86356. **(B)** Sample dendrogram and trait heatmap between control and myotonic dystrophy type 1 (DM1).

Supplementary Figure 3

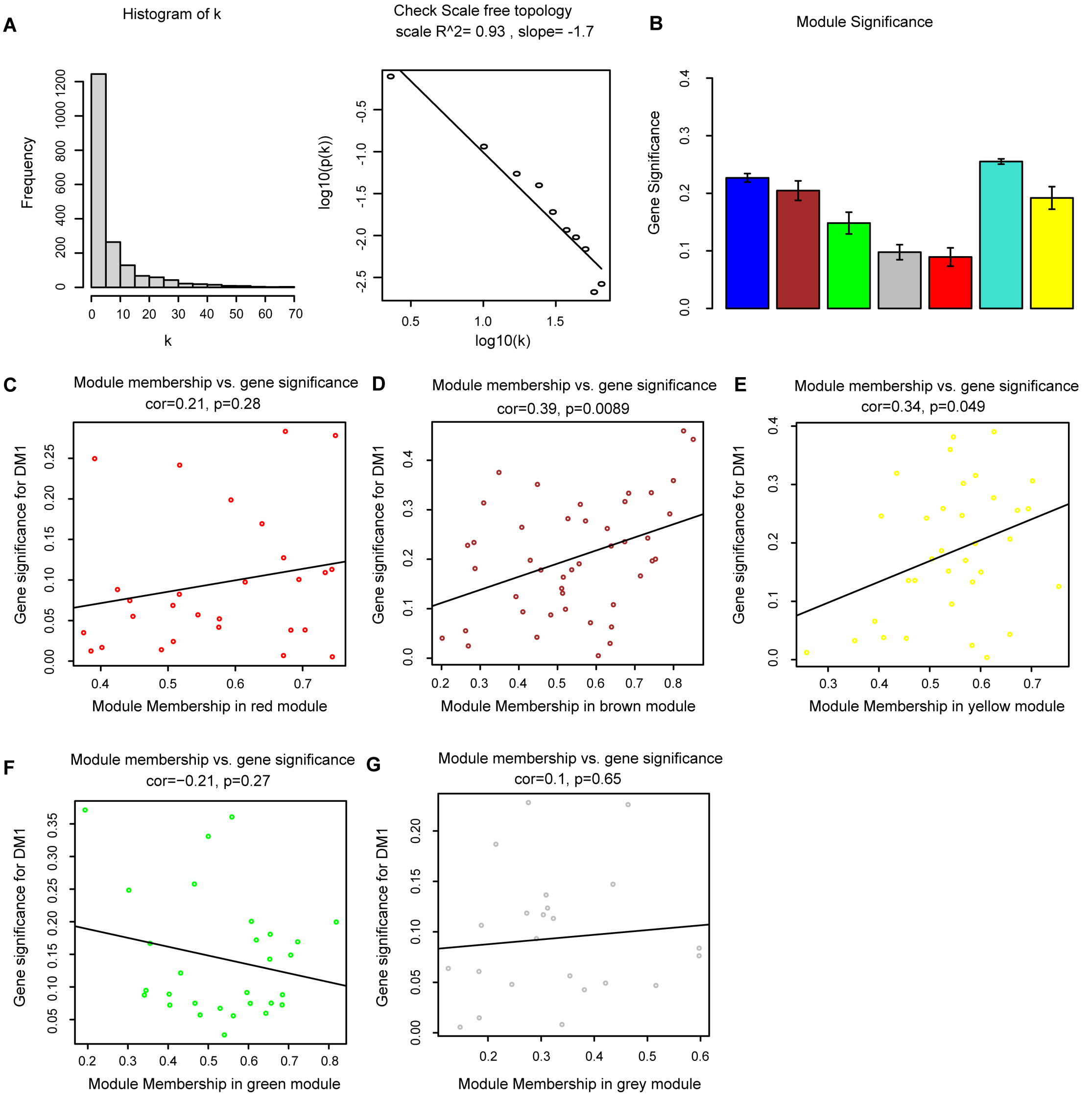

Supplementary Figure 3. Correlation of modules with DM1. (A) Histogram of connectivity distribution and determining the scale-free topology when $\beta = 5$. **(B)** Bar plot of correlation significance among the genes in WGCNA modules. **(C-G)** Scatterplot of gene significance (GS) for DM1 versus module membership (MM) in other modules.

Supplementary Figure 4

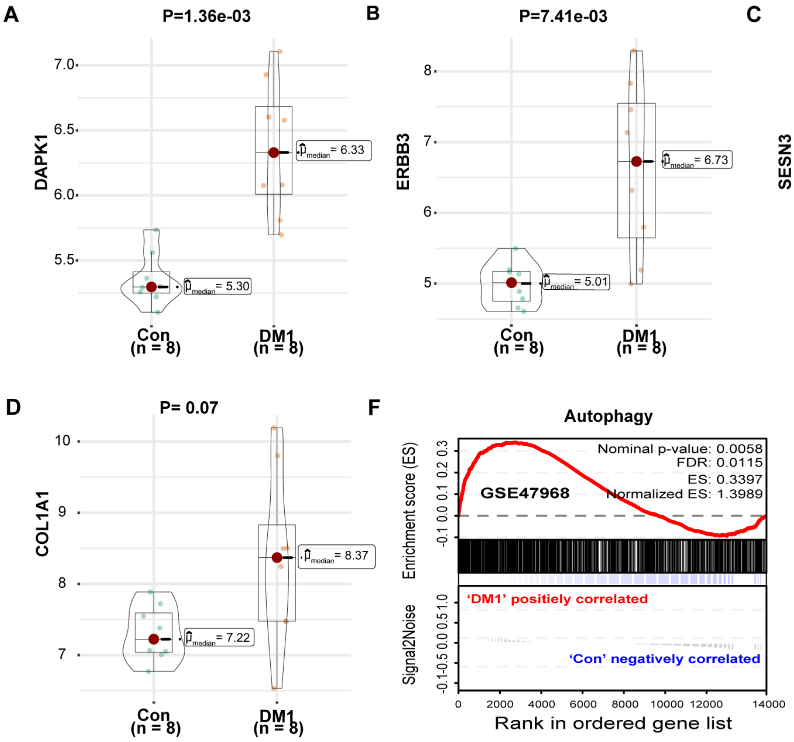

Supplementary Figure 4. Validation of hallmark genes in dataset GSE47968. (A–D) Expression levels of DAPK1, ERBB3, SESN3, and COL1A1 between DM1 and control. **(F)** GSEA result revealed significant enrichment of autophagy in DM1 based on the autophagy-related genes signature in GSE47968. Con, control; DM1, myotonic dystrophy type 1.

Supplementary Figure 5

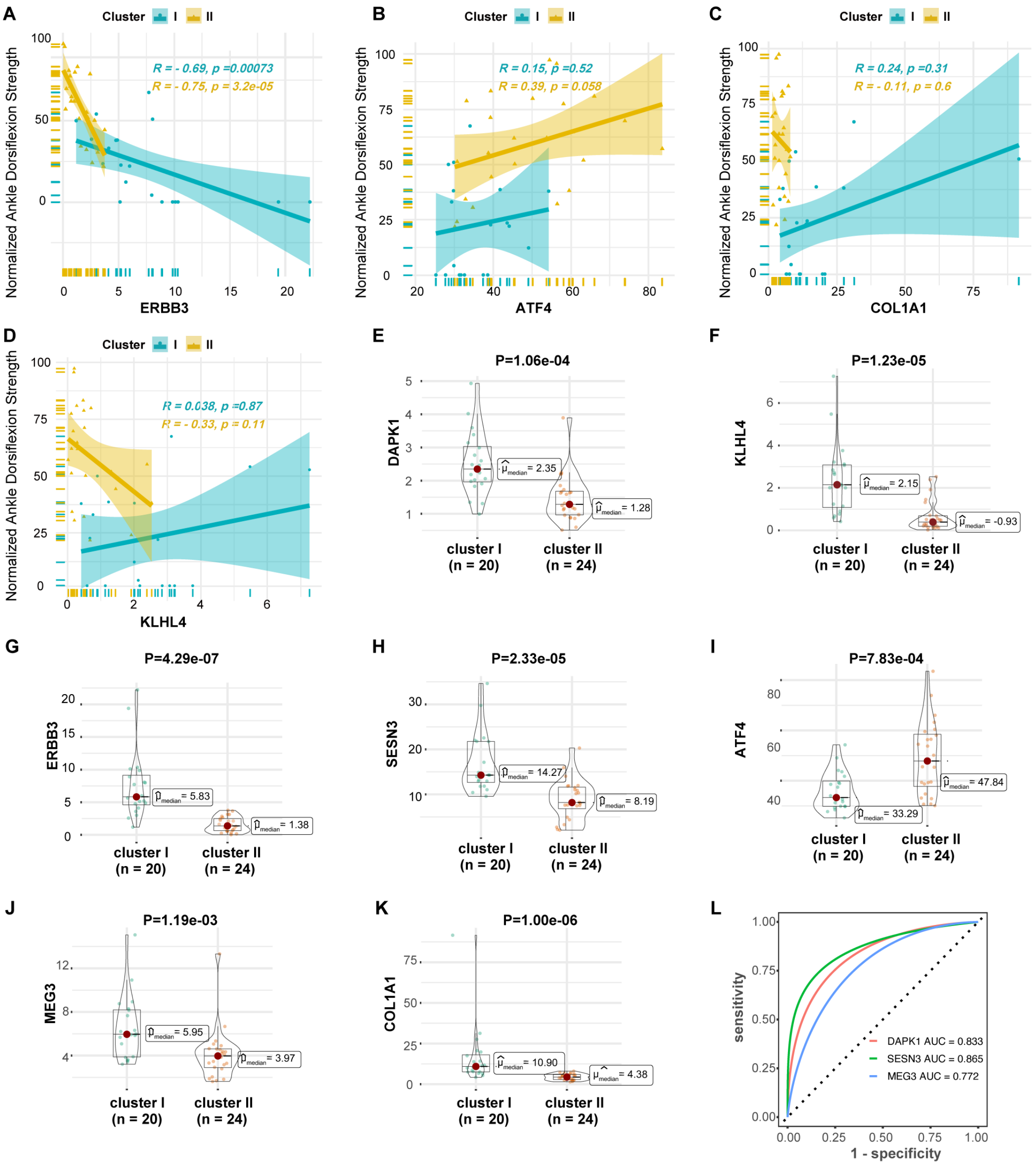

Supplementary Figure 5. Boxplot and GSEA visualization. (A-D) The correlation coefficients of gene expression relative to normalized ankle dorsiflexion strength between the two subgroups of DM1 was performed by line chart. **(E-K)** Boxplots show the expression level in DM1 subgroup of DAPK1, KLHL4, ERBB3, SESN3, ATF4, MEG3 and COL1A1. **(L)** ROC analysis for three hallmark genes (DAPK1, SESN3 and MEG3) expression of the two identified molecular clusters. Con, control; DM1, myotonic dystrophy type 1.
